# Associations of serum uric acid variability with neuroimaging metrics and cognitive decline: a population-based cohort study

**DOI:** 10.1186/s12916-024-03479-9

**Published:** 2024-06-20

**Authors:** Han Lv, Jing Sun, Tong Zhang, Ying Hui, Jing Li, Xinyu Zhao, Shuohua Chen, Wenjuan Liu, Xiaoshuai Li, Pengfei Zhao, Shouling Wu, Yanying Liu, Zhenchang Wang

**Affiliations:** 1 Department of Radiology, Beijing Friendship Hospital, Capital Medical University, Beijing, China; 2 Department of Rheumatology and Immunology, Beijing Friendship Hospital, Capital Medical University, Beijing, China; 3 Department of Radiology, Kailuan General Hospital, Hebei, Tangshan, China; 4 Department of Radiology, Beijing Tsinghua Changgung Hospital, School of Clinical Medicine, Tsinghua University, Beijing, China; 5 Clinical Epidemiology and Evidence-Based Medicine Unit, Beijing Friendship Hospital, Capital Medical University, Beijing, China; 6 Department of Cardiology, Kailuan General Hospital, Hebei, Tangshan, China; 7 Department of Medical Imaging, Aerospace Center Hospital, Beijing, China

**Keywords:** Cohort study, Serum uric acid, Variability, Magnetic resonance imaging, Brain health, Cognitive function

## Abstract

**Background:**

The relationship between variation in serum uric acid (SUA) levels and brain health is largely unknown. This study aimed to examine the associations of long-term variability in SUA levels with neuroimaging metrics and cognitive function.

**Methods:**

This study recruited 1111 participants aged 25–83 years from a multicenter, community-based cohort study. The SUA concentrations were measured every two years from 2006 to 2018. We measured the intraindividual SUA variability, including the direction and magnitude of change by calculating the slope value. The associations of SUA variability with neuroimaging markers (brain macrostructural volume, microstructural integrity, white matter hyperintensity, and the presence of cerebral small vessel disease) and cognitive function were examined using generalized linear models. Mediation analyses were performed to assess whether neuroimaging markers mediate the relationship between SUA variation and cognitive function.

**Results:**

Compared with the stable group, subjects with increased or decreased SUA levels were all featured by smaller brain white matter volume (beta =  − 0.25, 95% confidence interval [CI] − 0.39 to − 0.11 and beta =  − 0.15, 95% CI − 0.29 to − 0.02). Participants with progressively increased SUA exhibited widespread disrupted microstructural integrity, featured by lower global fractional anisotropy (beta =  − 0.24, 95% CI − 0.38 to − 0.10), higher mean diffusivity (beta = 0.16, 95% CI 0.04 to 0.28) and radial diffusivity (beta = 0.19, 95% CI 0.06 to 0.31). Elevated SUA was also associated with cognitive decline (beta =  − 0.18, 95% CI − 0.32 to − 0.04). White matter atrophy and impaired brain microstructural integrity mediated the impact of SUA increase on cognitive decline.

**Conclusions:**

It is the magnitude of SUA variation rather than the direction that plays a critical negative role in brain health, especially for participants with hyperuricemia. Smaller brain white matter volume and impaired microstructural integrity mediate the relationship between increased SUA level and cognitive function decline. Long-term stability of SUA level is recommended for maintaining brain health and preventing cognitive decline.

**Supplementary Information:**

The online version contains supplementary material available at 10.1186/s12916-024-03479-9.

## Background

Brain health is an evolving concept that is attracting increasing attention from both academic research community and from wider society [1]. Optimizing brain health improves physical and mental health and contributes to greater well-being of individuals across the life course. Not only cognitive function, but also the maintenance of optimal integrity in brain macro- and micro-structures, as well as the absence of neurological disease, are key features that delineate the concept of brain health [2].

Uric acid, as the final product of purine metabolism, is a naturally powerful antioxidant in human plasma. Several studies have demonstrated the neuroprotective properties of serum uric acid (SUA) towards dementia and cognitive dysfunction [3–5]. However, more recent researches suggest that such an association of SUA with cognitive functions may be U-shaped [6] or even negative [7–10]. For the integrity of brain structures and common pathological neurological processes, several studies reported that higher SUA levels were negatively associated with brain health, including decreased white matter (WM) volume [10], higher burden of WM hyperintensity (WMH) [11, 12], and presence of cerebral microbleeds (CMBs) [13]. However, the relationship between SUA concentration and brain health still remains underexplored.

The key issues may lie in the design and analytical methods of previous studies. In terms of study design, most prior research only collected SUA measurement at a single time point [14, 15], which may induce possible random errors. For the analytical methods, previous studies have not comprehensively analyzed the features of brain health from multiple aspects [11], particularly at the voxel level of macro- and micro-structures. Therefore, longitudinal follow-up data of SUA levels and multimodal neuroimaging data are in urgent need to comprehensively elucidate the impacts of SUA levels on brain health.

Acting as a potential neuroinflammation factor [16, 17], the alterations in SUA concentration may have a significant impact on the structural and functional integrity of the neural system. Previous research has demonstrated the significance of changes in physiological measurements on brain health [18, 19]. Similarly, laboratory assessments are susceptible to fluctuations, particularly in SUA levels. As a product of physiological metabolic processes, SUA levels are more prone to variations due to lifestyle factors [20, 21], and such variations are common in daily life. Large-scale cohort studies with long-term follow-up are essential for capturing the intraindividual fluctuations and changes of SUA concentrations over time through repeated measurements at multiple time points. However, the impacts of dynamic changes in SUA concentration on brain health are largely unknown.

In this study, we hypothesized that SUA variation, considering both its direction and magnitude of change during long-term follow-up, was associated with the alteration of brain health. The features of brain health will be assessed in terms of cerebral macro- and micro-structure and cognitive function. Multiple neuroimaging features will be analyzed at the voxel level. The mediating relationship between SUA variation, brain macro- and micro-structural impairment, and cognitive decline will be further discovered. The understanding of the integrated relationship between variations in SUA levels and a wide range of neuroimaging metrics and cognitive function will provide a novel perspective on the impact of SUA levels on brain health, thereby facilitating the development of innovative strategies for promoting brain health in the general population.

## Methods

### Study design and participants

This study originates from the Kailuan Study (KLS), which was conducted in the Kailuan community of Tangshan City, Northern China [22]. Initiated in 2006, the KLS is a population-based prospective cohort study to comprehensively evaluate the risk factors for all-cause mortality [23], cardiovascular diseases [24], and metabolic syndrome [25]. Demographic questionnaires and laboratory examinations were performed every 2 years according to standardized protocols at 11 local hospitals. Clinical data were recorded sequentially from 2006 to 2018, with a total of 7 visits.

Since December 2020, participants have been voluntarily recruited in the Multi-modality MEdical imaging sTudy bAsed on KLS (META-KLS), a subset of the KLS. Additional File 1 [22, 26–30] and Additional File 2: Fig. S1 provide a brief illustration of the META-KLS, and the detailed descriptions for this prospective cohort have been published recently [31]. Specifically, participants in the META-KLS voluntarily performed multi-modality brain magnetic resonance imaging (MRI) examinations to facilitate the assessment of brain health. As of September 2022, 1195 participants have completed brain MRI examinations for once. Moreover, it was worth noting that the participants in META-KLS were randomly selected from the KLS, thus the age and sex distribution among the participants were similar to those of the KLS [25, 30].

The inclusion criteria of this study were as follows: (1) SUA and other clinical parameter measurements were acquired more than three times of follow-up; (2) completed one brain MRI examination during 2020 to 2022; and (3) absence of clinically diagnosed cardiovascular disease, stroke, dementia, or neuropsychiatric disease. The exclusion criteria were (1) missing brain MRI data; (2) missing age and (or) sex information; and (3) a known history of cancer.

This study follows the Strengthening the Reporting of Observational Studies in Epidemiology statement [32].

### Measurements of clinical features

After fasting overnight, blood samples were collected from the anterior elbow vein and infused into a vacuum tube containing ethylenediamine tetraacetic acid (EDTA). The concentration of SUA in pre-treated blood samples was detected using a commercial kit (Ke Hua Biological Engineering Corporation, Shanghai, China) and an automated biochemical analyzer (Hitachi 7600, Tokyo, Japan). The uricase method was used for SUA measurement over the entire follow-up time.

Measurements of other clinical features are listed in Additional File 1. Recorded data including body mass index, smoking habits, habitual alcohol consumption, physical activity routines, history of hypertension, history of diabetes, total cholesterol, triglyceride, high-density lipoprotein cholesterol, and low-density lipoprotein cholesterol.

### Serum uric acid variability

The intraindividual variability of SUA concentrations was the primary exposure in this study. For the assessment of SUA variability, we developed a general linear model by incorporating all SUA measurements of each participant from 2006 to 2018 to calculate the slope value. The least squares method was employed to determine the slope, which reflect both the direction and magnitude of SUA change over the follow-up period. The slope calculated using the least squares method is a well-established technique for trend detection. This approach takes into account all the measurements of each participant, allowing for an assessment of variability within each individual.

We categorized SUA variability into three groups based on the calculated slope value. The increased group was defined as the top 20% of all slope values (> 8.88 μmol/L/2 years), while the decrease group was defined as the bottom 20% of slope values (< − 1.04 μmol/L/2 years). The remaining participants were defined as the stable group, which was set as a reference.

### Neuroimaging metrics of brain health

Neuroimaging data were acquired using a 3.0-Tesla MRI scanner (General Electric 750W, Milwaukee, WI, USA). According to the META-KLS protocol, standardized sequences included three-dimensional (3D) brain volume (BRAVO) for brain macrostructural volume analysis based on high-resolution T1-weighted imaging (T1WI), diffusion tensor imaging (DTI) for brain microstructural integrity analysis, 3D fluid-attenuated inversion recovery (FLAIR) for WMH analysis, T2-weighted imaging and susceptibility-weighted angiography for cerebral small vessel disease (CSVD) evaluation, and diffusion-weighted imaging for the determination of ischemic stroke [31]. The parameters were listed in Additional File 3: Table S1. Decreased brain macrostructural volume, impaired microstructural integrity, higher volume of WMH, or the presence of CSVD have been suggested to be associated with worse brain health [18, 33].

#### Brain macrostructural volume

The total intracranial volume (TIV), the volumes of brain gray matter (GM), white matter (WM), and cerebrospinal fluid (CSF) were quantified automatically using Statistical Parametric Mapping software (http://www.fil.ion.ucl.ac.uk/spm) based on 3D-BRAVO-T1WI sequence. The volume of supratentorial cerebral parenchyma was defined as the sum of GM and WM volume.

These absolute volumetric measurements were further calculated as the percentage of TIV to normalize for head size. A relatively small volume of the GM, WM, or larger volume of CSF indicates brain macrostructural atrophy. The results were then z-transformed. We also analyzed the GM and WM differences between the groups at the voxel level.

#### Brain microstructural integrity

To evaluate early-stage changes in cerebral white matter, we measured the global fractional anisotropy (FA), mean diffusivity (MD), axial diffusivity (AD), and radial diffusivity (RD) based on DTI sequence to reflect white matter microstructural integrity. Specifically, FA indicates the coherence of directionality of water molecule diffusion. AD and RD measure the magnitude of water molecule diffusion in different directions. MD represents the average diffusion that is unrelated to tissue-based directionality, while AD and RD assess axonal and myelin integrity, respectively [34, 35]. In general, decreased FA and increased MD, AD, or RD value implicate the worse microstructural integrity. The quantification results were also z-transformed. Additionally, tract-based spatial statistics analyses were performed to analyze the difference in skeletonized microstructural integrity between the groups.

#### White matter hyperintensity

The WMH was exhibited as white matter lesions with increased brightness compared to adjacent normal brain tissue on the 3D-FLAIR sequence. Volumetric results, including total WMH, periventricular WMH (PWMH), and deep WMH (DWMH), were further calculated as the percentage of TIV for normalization and z-transformed.

#### Cerebral small vessel disease

The presence of CSVD and its imaging markers were assessed by two well-trained neuroradiologists with 12 years of experience and further confirmed by a third neuroradiologist with 10 years of experience, all of whom were blinded to the participants’ clinical information. We recorded the presence of CSVD and four imaging markers including CMBs, moderate-to-severe basal ganglia enlarged perivascular spaces (BG-EPVS), lacune, and WMH burden [36, 37]. According to the Fazekas scale, the WMH burden was evaluated based on the expanding range of PWMH and DWMH (PWMH Fazekas 3 or DWMH Fazekas 2–3) [38]. The total CSVD burden was rated from 0 to 4 according to the widely accepted Wardlaw group method [37]. One point was assigned for the following four imaging manifestations separately: WMH burden, presence of lacune or CMB, or moderate to severe BG-EPVS (*N* > 10) [37].

### Cognitive function assessment

A face-to-face questionnaire survey was conducted by a senior psychiatrist in Kailuan Mental Health Center on the day of neuroimaging data acquisition. Subjects were evaluated using the Montreal Cognitive Assessment (MoCA) with a maximum score of 30 points and seven cognitive domains [39]. To adjust for educational bias, one point was added to the total score for those with fewer than 12 years of education. A final score of 25 points or less indicates cognitive impairment. The final total scores were also transformed into *z*-scores.

### Statistical analysis

We applied generalized linear models to analyze the relationship between SUA variability and relative brain macrostructural volume, microstructural integrity, relative volume of white matter hyperintensity, CSVD markers, and MoCA scores. Age, sex, body mass index, smoking habits, habitual alcohol consumption, physical activity routines, history of hypertension, history of diabetes, total cholesterol, triglycerides, high-density lipoprotein cholesterol, and low-density lipoprotein cholesterol were added as covariates to adjust for potential confounding. Microstructural integrity analysis was further adjusted for WM and WMH volume, and MoCA scores were further adjusted for the proportion of WMH volume to TIV. The significance threshold was set at 2-sided *p*-value < 0.05 determined by the least significance difference considered statistically significant, after correcting for multiple testing.

For the potential nonlinear association between SUA variability and neuroimaging metrics, we further conducted the generalized additive model to explore the presence of a nonlinear U-shaped relationship. Sensitivity analyses were performed in the subgroups stratified by age and sex separately to examine the robustness and consistency of the main results. The cut-off points for age were 45 and 60 years. Additionally, all participants were divided into non-hyperuricemia and hyperuricemia subgroups, with SUA > 360 μmol/L in women and > 420 μmol/L in men defined as hyperuricemia [40]. We also performed sensitivity analyses in non-hyperuricemia and hyperuricemia participants. SPSS Statistics 27 (IBM Corp., Armonk, NY, USA) and R 4.2.2 (R Development Core Team) were used for statistical analysis.

For neuroimaging analysis at the voxel-wise level, the general linear model was applied using the randomize option in FMRIB Software Library (FSL v6.0) to analyze differences in brain tissue volumes and microstructural integrity. Significant clusters were determined by employing threshold-free cluster enhancement (TFCE) via 5000 permutations. We reported regions of *p*_TFCE_ < 0.01 with age, sex, and TIV as covariates for GM and WM volume changes. Clusters with *p*_TFCE_ < 0.01 and probabilities of affected tracts threshold > 1% were reported for microstructural integrity changes, with age and sex as covariates.

We used the PROCESS procedure to assess whether the brain MRI markers mediated the association between SUA variance and cognitive function. The total, direct, and indirect effects were estimated based on 5000 bootstrap samples.

## Results

In total, 1111 participants aged 25 to 83 years were eligible for analysis (Additional File 2: Fig. S2). The mean age was 55.2 ± 11.5 years, and female participants accounted for 44.6% (496 of 1111). Table 1 shows the demographic and clinical characteristics of included participants. Table 2 summarizes the characteristics of neuroimaging metrics.

**Table 1:** Demographic and clinical characteristics of the participants

Variables	Overall
Age, median (IQR), years^a^	56.0 (47.0, 65.0)
Female, No. (%)	496 (44.6)
Serum uric acid at baseline, median (IQR), μmol/L	284.0 (229.0, 347.0)
Slope value, median (IQR), μmol/L/2 years	3.7 (− 0.1, 7.6)
Body mass index at baseline, median (IQR), kg/m^2^	24.5 (22.4, 26.9)
Current smoking, No. (%)	225 (20.3)
Current drinking, No. (%)	297 (26.7)
Usually physical activity, No. (%)	545 (49.1)
History of hypertension, No. (%)	539 (48.8)
Blood pressure at baseline, median (IQR), mm Hg	
Systolic	119.3 (109.3, 129.3)
Diastolic	79.3 (70.0, 81.3)
History of diabetes, No. (%)	213 (19.2)
Cholesterol at baseline, mmol/L	
Total cholesterol, median (IQR)	4.7 (4.1, 5.4)
Triglyceride, median (IQR)	1.2 (0.8, 1.8)
High density lipoprotein cholesterol, median (IQR)	1.5 (1.3, 1.7)
Low density lipoprotein cholesterol, median (IQR)	2.2 (1.8, 2.6)
MoCA score, median (IQR)	25 (23, 27)

Values are presented as median (interquartile range) and *No*. (%)

Abbreviation: *No* number, *IQR* interquartile range, *MoCA* Montreal Cognitive Assessment

^a^Age is calculated at the time of magnetic resonance imaging acquisition

**Table 2:** Description of brain magnetic resonance imaging markers of the participants

Brain MRI marker	No. of participants	Overall
TIV, mean (SD), ml	1,010	1498.7 (139.4)
Brain macrostructural volume, mean (SD), % of TIV	1,010	
Brain parenchyma		73.3 (4.1)
Gray matter		39.9 (2.7)
White matter		33.4 (2.1)
Cerebrospinal fluid		26.5 (4.1)
Brain microstructural integrity, mean (SD)	1,040	
Fractional anisotropy		0.46 (0.03)
Mean diffusivity, 10^−3^ mm^2^/s		0.82 (0.04)
Axial diffusivity, 10^−3^ mm^2^/s		1.27 (0.03)
Radial diffusivity, 10^−3^ mm^2^/s		0.60 (0.04)
White matter hyperintensity, median (IQR), % of TIV	985	
White matter hyperintensity		0.23 (0.11–0.55)
Periventricular white matter hyperintensity		0.14 (0.06–0.33)
Deep white matter hyperintensity volume		0.09 (0.03–0.22)
Cerebral small vessel disease, No. (%)	1,012	
Presence of cerebral small vessel disease		700 (69.2)
Presence of cerebral microbleeds		278 (27.5)
Presence of moderate-to-severe basal ganglia enlarged perivascular spaces		613 (60.6)
Presence of lacune		172 (17.0)
Presence of white matter hyperintensity burden		284 (28.1)

Values are presented as mean (standard deviation), median (interquartile range), or No. (%)

Abbreviations: *SD *standard deviation*, IQR *interquartile range*, No *number*, TIV* total intracranial volume, *MRI* magnetic resonance imaging

### Association between SUA variability and brain health

**Fig. 1:**
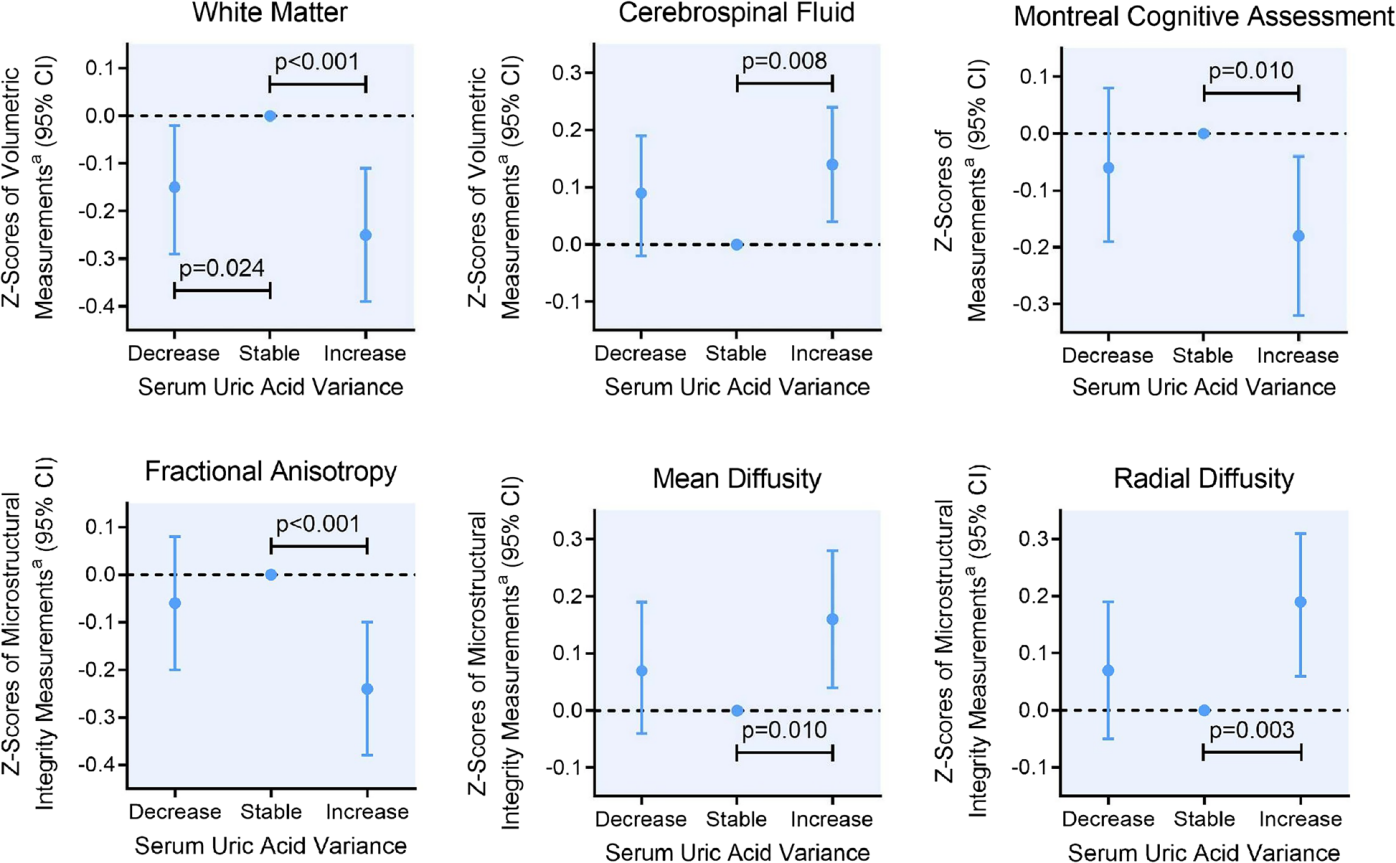
Association between serum uric acid variability and brain macrostructural volume and microstructural integrity. Abbreviation: CI, confidence interval. ^a^Covariates included age, sex, body mass index, smoking habits, alcohol habits, physical activity habits, history of hypertension, history of diabetes, total cholesterol, triglycerides, high-density lipoprotein cholesterol, and low-density lipoprotein cholesterol

Figure 1 and Table 3 show the multivariable-adjusted association between SUA variability and brain tissue volumes and microstructural integrity. Both the SUA increase and decrease groups exhibited smaller WM volume (beta =  − 0.25, 95% confidence interval [CI] − 0.39 to − 0.11, *p* value < 0.001; beta =  − 0.15, 95% CI − 0.29 to − 0.02, *p* value = 0.024). The analysis of generalized additive model was performed by setting the SUA slope value for each participant as an independent variable. The results revealed a significantly nonlinear U-shaped relationship between SUA slope and WM volume, with an edf value of 5.036 and a *p* value of 0.0159.

**Table 3:** Association between serum uric acid variability and brain macrostructural volume, microstructural integrity, white matter hyperintensity, cerebral small vessel disease, and cognitive function

Index of brain health	Serum uric acid variance, μmol/L/2 years				
	**Decrease**	***p* value**	**Stable**	**Increase**	***P* value**
**Brain macrostructural volume, % of TIV (in *z*-score)**	***N* = 204**		***N* = 608**	***N* = 195**	
Cerebral parenchyma^a^	− 0.08 (− 0.18 to 0.02)	0.098	0 (ref)	** − 0.14 (− 0.24 to − 0.04)**	**0.008**
Gray matter^a^	− 0.01 (− 0.11 to 0.09)	0.856	0 (ref)	− 0.02 (− 0.12 to 0.09)	0.730
White matter^a^	** − 0.15 (− 0.29 to − 0.02)**	**0.024**	0 (ref)	** − 0.25 (− 0.39 to − 0.11)**	** < 0.001**
Cerebrospinal fluid^a^	0.09 (− 0.02 to 0.19)	0.095	0 (ref)	**0.14 (0.04 to 0.24)**	**0.008**
**Brain microstructural integrity (in *z*-score)**	***N* = 206**		***N* = 624**	***N* = 204**	
Fractional anisotropy^a^	− 0.06 (− 0.20 to 0.08)	0.415	0 (ref)	** − 0.24 (− 0.38 to − 0.10)**	** < 0.001**
Mean diffusivity^a^	0.07 (− 0.04 to 0.19)	0.221	0 (ref)	**0.16 (0.04 to 0.28)**	**0.010**
Axial diffusivity^a^	0.07 (− 0.05 to 0.19)	0.243	0 (ref)	0.06 (− 0.07 to 0.18)	0.370
Radial diffusivity^a^	0.07 (− 0.05 to 0.19)	0.251	0 (ref)	**0.19 (0.06 to 0.31)**	**0.003**
Fractional anisotropy^b^	− 0.02 (− 0.15 to 0.12)	0.779	0 (ref)	** − 0.20 (− 0.33 to − 0.06)**	**0.006**
Mean diffusivity^b^	0.04 (− 0.07 to 0.15)	0.455	0 (ref)	**0.13 (0.02 to 0.24)**	**0.022**
Axial diffusivity^b^	0.05 (− 0.06 to 0.16)	0.365	0 (ref)	0.04 (− 0.07 to 0.16)	0.461
Radial diffusivity^b^	0.04 (− 0.08 to 0.15)	0.535	0 (ref)	**0.15 (0.04 to 0.27)**	**0.008**
**White matter hyperintensity, % of TIV (in z-score)**	***N* = 196**		***N* = 596**	***N* = 191**	
White matter hyperintensity^a^	0.07 (− 0.07 to 0.20)	0.336	0 (ref)	0.09(− 0.05 to 0.22)	0.228
Periventricular white matter hyperintensity^a^	0.06 (− 0.07 to 0.19)	0.367	0 (ref)	0.10 (− 0.03 to 0.23)	0.134
Deep white matter hyperintensity^a^	0.07 (− 0.08 to 0.21)	0.366	0 (ref)	0.07 (− 0.08 to 0.21)	0.366
**Cerebral small vessel disease**	***N* = 205**		***N* = 610**	***N* = 192**	
Presence of cerebral small vessel disease^a^	1.58 (1.00 to 2.51)	0.050	1 (ref)	1.14 (0.72 to 1.79)	0.585
Presence of cerebral microbleeds^a^	0.90 (0.61 to 1.32)	0.591	1 (ref)	0.74 (0.49 to 1.12)	0.154
Presence of moderate-to-severe basal ganglia enlarged perivascular spaces^a^	1.31 (0.86 to 2.01)	0.207	1 (ref)	1.03 (0.68 to 1.58)	0.886
Presence of lacune^a^	0.92 (0.56 to 1.51)	0.747	1 (ref)	1.03 (0.63 to 1.69)	0.919
Presence of white matter hyperintensity burden^a^	0.94 (0.62 to 1.42)	0.768	1 (ref)	0.95 (0.62 to 1.45)	0.794
**Cognitive assessment (in z-score)**	***N* = 214**		***N* = 653**	***N* = 218**	
MoCA scores^a^	− 0.06 (− 0.19 to 0.08)	0.408	0 (ref)	** − 0.18 (− 0.32 to − 0.04)**	**0.010**
MoCA scores^c^	− 0.04 (− 0.18 to 0.11)	0.610	0 (ref)	** − 0.20 (− 0.34 to − 0.05)**	**0.009**

Abbreviations: *SUA*, serum uric acid; *MoCA*, Montreal Cognitive Assessment

^a^Covariates included age, sex, smoking habits, habitual alcohol consumption, physical activity routines, body mass index, history of hypertension, history of diabetes, total cholesterol, triglyceride, high-density lipoprotein cholesterol, and low-density lipoprotein cholesterol

^b^With further adjustment for white matter and white matter hyperintensity volume

^c^With further adjustment for the proportion of white matter hyperintensity volume to total intracranial volume

Compared to the stable group, the increase in SUA was also associated with smaller cerebral parenchyma volume (beta =  − 0.14, 95% CI − 0.24 to − 0.04, *p* value = 0.008) and larger CSF volume (beta = 0.14, 95% CI 0.04 to 0.24, *p* value = 0.008). Participants with progressively increased SUA levels also showed lower FA (beta =  − 0.24, 95% CI − 0.38 to − 0.10, *p* value < 0.001), higher MD (beta = 0.16, 95% CI 0.04 to 0.28, *p* value = 0.010), and higher RD value (beta = 0.19, 95% CI 0.06 to 0.31, *p* value = 0.003). The associations for FA, MD, and RD measures remained significant after further adjustment for WM and WMH volume. These associations presented primarily in elder participants (aged > 60 years) (Additional File 3: Table S2) and male participants (Additional File 3: Table S3).

The elevation in SUA levels during follow-up was also associated with lower MoCA scores (beta =  − 0.18, 95% CI − 0.32 to − 0.04, *p* value = 0.010). This result remained significant after additional adjustment for WMH volume (Table 3). The association between SUA variance and the presence of CSVD reached marginal significance. We did not observe a significant association between SUA variance and WMH volume (Table 3).

In the sensitivity analysis with non-hyperuricemia participants, we observed similar significant associations between SUA increase and smaller WM volume, lower FA value, and lower MoCA scores. For the remaining main findings, the associations did not reach statistical significance in the non-hyperuricemia group, but the consistent trends were detected (Additional File 3: Table S4). The subgroup analyses with hyperuricemia participants also yielded similar findings to the main results. Furthermore, decreased SUA variation was associated with smaller cerebral parenchyma volume (beta =  − 0.28, 95% CI − 0.55 to − 0.01, *p* value = 0.042), larger CSF volume (beta = 0.29, 95% CI 0.02 to 0.57, *p* value = 0.034), as well as lower FA value (beta =  − 0.38, 95% CI − 0.68 to − 0.09, *p* value = 0.011) (Additional File 3: Table S5).

**Fig. 2:**
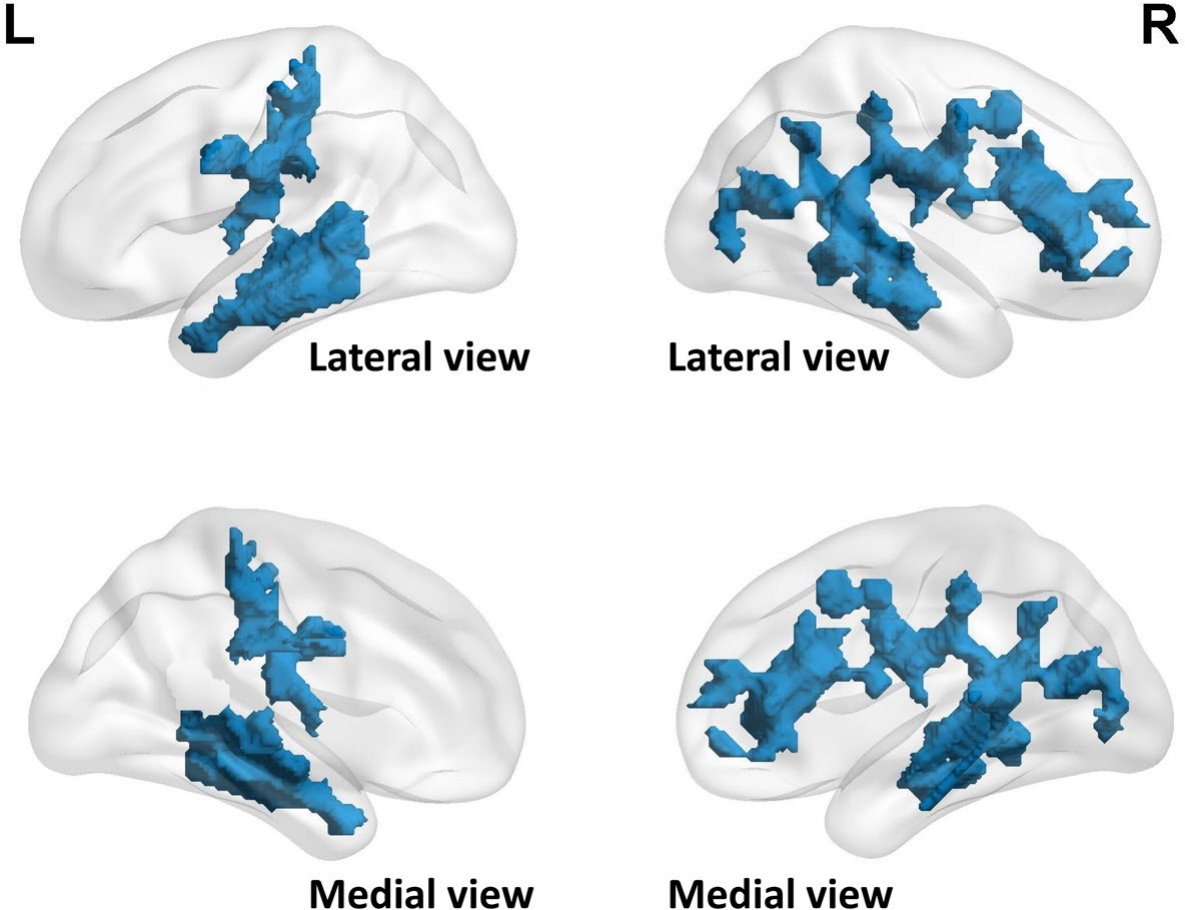
Association between serum uric acid variability and brain macrostructural volume in brain subregions at the voxel level. Abbreviations: L, left; R, right. Results are comparisons between the increased group and stable group. Regions of *p*_threshold-free cluster enhancement_ < 0.01 for white matter volume changes are reported. Cold color represents decreased values. Covariates include age, sex, and total intracranial volume

**Fig. 3:**
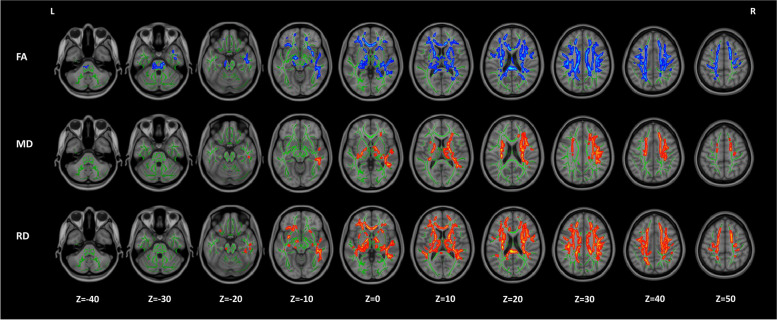
Association between serum uric acid variability and brain microstructural integrity in brain subregions at the voxel level. Abbreviations: FA, fractional anisotropy; MD, mean diffusivity; RD, radial diffusivity; L, left; R, right. Results are comparisons between the increased group and stable group. Clusters with *p*_threshold-free cluster enhancement_ < 0.01 are demonstrated. Covariates include age and sex. Hot and cold colors represent increased and decreased values, respectively

Additional analysis at the voxel-wise level did not reveal significant alteration in GM volume in the increased group compared to the stable group. However, relatively smaller volume in widespread regions of WM, mainly in the frontal and temporal lobes, indicated that alterations in cerebral parenchyma may be driven by WM atrophy for the SUA increased group (Fig. 2). Analysis of microstructural integrity at the voxel level demonstrated widespread disruptions in FA, MD, and RD in the increased group (Fig. 3 and Additional File 3: Table S6).

### Association between neuroimaging metrics and cognitive function

Generalized linear models revealed that WM volumes and cerebral parenchyma volumes were positively associated with higher MoCA scores (beta = 0.12, 95% CI 0.05 to 0.18, *p* value < 0.001; beta = 0.16, 95% CI 0.07 to 0.25, *p* value < 0.001). For microstructural analysis, the FA values were also associated with higher MoCA scores (beta = 0.12, 95% CI 0.06 to 0.18, *p* value < 0.001), while the MD and RD values associated with lower MoCA scores (beta =  − 0.18, 95% CI − 0.25 to − 0.11, *p* value < 0.001; beta =  − 0.17, 95% CI − 0.24 to − 0.10, *p* value < 0.001) (Additional File 3: Table S7).

### Mediating effects of neuroimaging metrics on the relationship between SUA variance and cognitive function

**Fig. 4:**
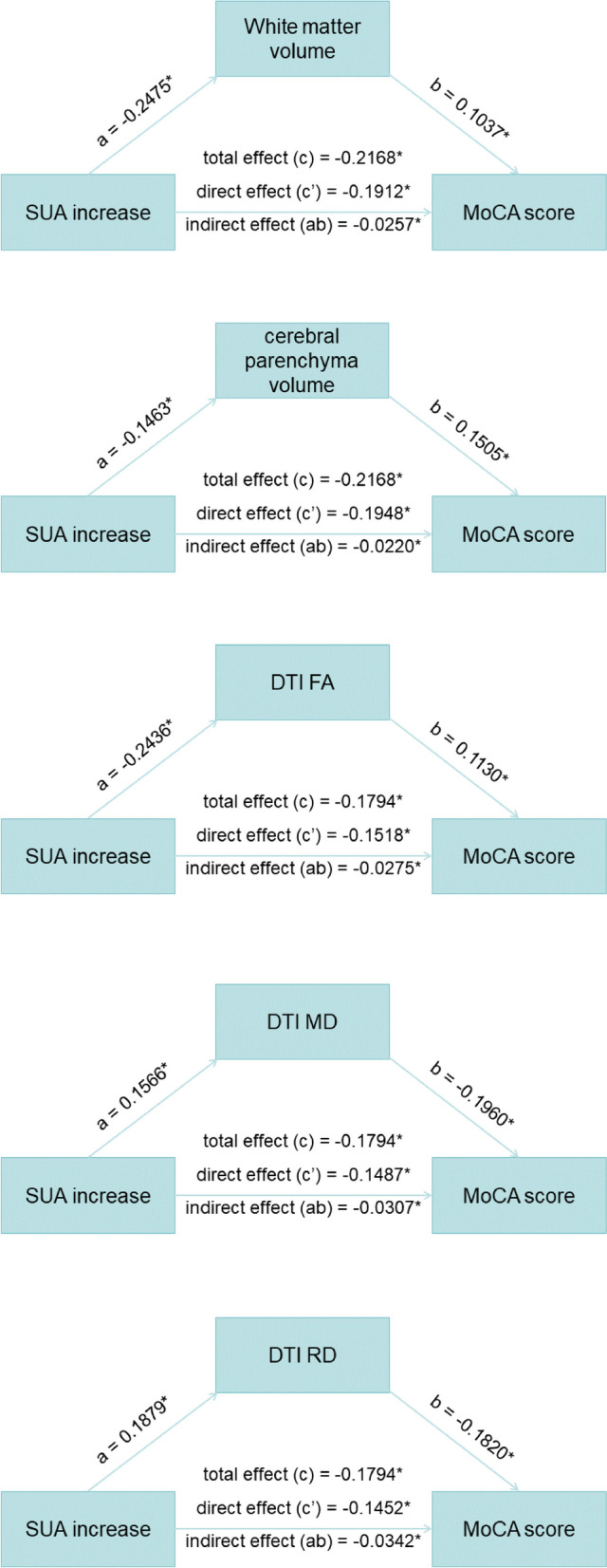
Mediation effect by cerebral macro- and micro-structural impairment in the association between serum uric acid variability and cognitive decline. Abbreviations: SUA, serum uric acid; MoCA, Montreal Cognitive Assessment; DTI, diffusion tensor imaging; FA, fractional anisotropy, MD, mean diffusivity; RD, radial diffusivity. ^*^The statistical significance threshold was set at *p* < 0.05

The mediation analyses were conducted with SUA stable or increase as the independent variable *X*, and the z-transformed MoCA scores as the dependent variable *Y*. The results showed that the effects of increased SUA on lower MoCA scores were partially mediated by decreased WM and cerebral parenchyma volumes, lower FA, and higher MD and RD values (Fig. 4). The proportions of mediating effects were 11.85%, 10.15%, 15.33%, 17.11%, and 19.06%, respectively.

## Discussion

This cohort study with 16-year follow-up examined the associations between variability in SUA levels and multimodal neuroimaging metrics and cognitive function. The primary findings suggest that SUA variation, especially progressively increased SUA levels during long-term follow-up, was a critical risk factor for negative changes of brain health. Specifically, subjects with progressively elevated SUA levels were featured by brain tissue atrophy, disrupted microstructural integrity, and poor cognitive performance compared to those with stable SUA levels. The decreased SUA levels were also associated with brain tissue atrophy. In addition, in participants with hyperuricemia, the associations with brain tissue atrophy and disrupted microstructural integrity were observed for both falls and rises in SUA levels. The findings suggest that it is the magnitude of SUA variation rather than the direction (i.e., rise or fall) that plays a critical negative role in brain health, especially for participants with hyperuricemia. We also elucidated the mediating role of brain tissue atrophy and microstructural damage in the relationship between SUA elevation and cognitive decline.

In clinical practice, healthcare providers generally monitor the concentration of SUA as part of routine assessments. To our knowledge, this population-based cohort study proposes for the first time that variations in SUA levels have a significant detrimental impact on brain structures. This suggests that long-term fluctuations in SUA concentration can adversely affect brain health. Therefore, our findings indicate that clinicians should pay more attention to the changes in SUA levels and emphasize the importance of maintaining the long-term stability of SUA for preserving brain health. In clinical practice and daily life, the SUA levels can be influenced by a variety of factors, such as metabolic disorders [41], medications [42, 43], and lifestyles [44, 45]. For the general population, it is recommended to have regular physical examinations and adopt a healthy lifestyle and dietary habits to sustain relatively stable SUA levels, thereby promoting brain health.

There are several advantages of this study. The major strength of this study lies in the repeated measurements of SUA levels, which could reduce random errors that may be present in studies with a cross-sectional design. The large-sample cohort study with long-term follow-up enables a comprehensive investigation of the relationship between SUA variability, considering both the direction and magnitude of variation, and brain structures as well as cognitive function, thus providing a novel perspective on the impact of SUA levels on brain health. Second, multi-modality brain MRI acquisition also allows for the accurate and comprehensive assessment of brain health. Notably, our study also examined the association at the voxel level, revealing the effect of SUA change on brain sub-regional structures precisely. Third, when we analyze the features of brain microstructural integrity, results were further adjusted for WM and WMH to exclude possible impacts from WM structure. We also adjusted relative WMH volume to exclude possible effects of WM lesions on cognitive function. Results remained significant after adjustment, proving the robustness of the analysis. Fourth, through the mediation analysis, we demonstrated the unidirectional associations that the elevation in SUA levels affects cognitive decline through impairment to brain macro- and micro-structures. In addition, the large amount of subjects with a wide range of ages in META-KLS was representative, as the subjects were enrolled from multiple hospitals of Tangshan in the center of the Bohai Sea Gulf region. The results may be well generalized to Northern Chinese people.

The relationship between SUA levels and cognitive function has been long debated [3, 6, 10, 46]. Most studies reported a negative association between high SUA levels and cognitive performance [8, 10, 46–48]. However, several studies observed positive associations [3, 49] or U-shaped associations [6]. Limited by study designs, previous studies could only analyze the effect of SUA at the same time the cognitive function was evaluated. This study, however, analyzed the associations between SUA levels and cognitive function from a totally different aspect. Supported by the repeated SUA measurements at multiple time points, our study reported for the first time that progressively elevated SUA levels during long-term follow-up are the critical factor that detrimentally affects cognitive function. This finding highlights the negative effect of SUA variation on cognitive function.

A previous cross-sectional study based on the Rotterdam Study revealed that single-time measured higher SUA levels were associated with smaller total WM volume, but not with GM volume [10]. Similar to this finding, our study also found a significant association with brain volume only in the WM, regardless of global or regional white matter volumes. In addition, the highlight of this study lies in that we repeatedly collected SUA data biennially over a 12-year follow-up period. The availability of repeated measurements for SUA concentrations can reduce random errors and capture the intraindividual fluctuations and changes of SUA over time, which are not feasible in previous investigations on this topic. Thus, the design of our cohort study provides a robust framework for investigating the associations of long-term variability in SUA levels with brain structures, thereby enabling us to gain deeper insights into the nature of the relationship between SUA levels and brain structural features. Firstly, both progressively increased and decreased SUA levels are associated with WM atrophy, suggesting that it is the magnitude of SUA variation rather than the direction (i.e., rise or fall) that plays a critical role. Secondly, we found that the frontal and temporal lobes are particularly susceptible to elevated SUA levels. Thirdly, not only WM atrophy, but also features of WM microstructural integrity (lower FA, and higher MD and RD values) are key factors mediating cognitive decline. In summary, these results suggest that WM is more likely to be affected by changes of SUA level, highlighting the importance of preventing long-term fluctuation in SUA throughout life, rather than simply lowering the SUA level, for better brain health and preservation of cognitive function.

The underlying mechanisms for the impact of SUA variation on brain health have not been well-elucidated. This association may be mediated by potential chronic subclinical neuroinflammation induced by SUA change [50]. A persistently responsive immune system is present regardless of the SUA level [51]. However, the immune response may fail to adapt in response to progressively elevated SUA levels over the years. Alternatively, SUA may reduce cell viability, increase oxidative stress, promote lipid peroxidation and apoptosis of vascular smooth muscle cells, induce endothelial dysfunction, and potentiate the proapoptotic effect of amyloid β [47, 52]. The combined effect of these factors can damage the microvascular endothelium and destroy the blood–brain barrier. Progressively increasing SUA levels may also enhance this deteriorating effect [53]. The possible mediating effects of different inflammatory biomarkers warrant further investigation.

Several limitations in the present study also need to be considered. First, only one brain MRI examination and one assessment of cognitive function were conducted, which does not allow for the determination of causality between SUA levels and brain structural changes or cognitive outcomes. Longitudinal brain MRI data and cognitive assessments at multiple time points are warranted in the future to analyze the dynamic changes in brain health status. Second, two subjects were excluded from the analysis due to the absence of neuroimaging data. Moreover, as the subjects were randomly selected from the Kailuan Study and voluntarily underwent brain MRI examinations, there may be some degree of potential selection bias. Third, while the 16-year follow-up period in this study offers valuable insights into the long-term impacts of SUA variability on brain health, these findings need to be interpreted with caution in the context of different follow-up periods. Additionally, while biennial follow-up can provide insight into the trend of variability in SUA levels, it may not sufficiently capture the day-to-day fluctuations.

## Conclusions

It is the magnitude of SUA variation rather than the direction that plays a critical negative role in brain health, especially for participants with hyperuricemia. Smaller cerebral parenchyma volume, WM atrophy, and impaired microstructural integrity mediate the association between increased SUA levels and cognitive decline. Long-term stability of SUA level is essential for maintaining brain health and preventing cognitive decline.

### Supplementary Information

Additional file 1. Supplementary Methods.

Additional file 2: Figures S1-S2. Fig. S1. Schematic diagram of the study. Fig. S2. Flowchart of included participants.

Additional file 3: Tables S1-S7. Tables S1. Detailed neuroimaging scanning parameters.  Tables S2. Serum uric acid variability, brain macrostructural volume, microstructural integrity, white matter hyperintensity, cerebral small vessel disease, and cognitive function stratified by age.  Tables S3. Serum uric acid variability, brain macrostructural volume, microstructural integrity, white matter hyperintensity, cerebral small vessel disease, and cognitive function stratified by sex. Tables S4. Serum uric acid variability, brain macrostructural volume, microstructural integrity, white matter hyperintensity, cerebral small vessel disease, and cognitive function among non-hyperuricemic populations. Tables S5. Serum uric acid variability, brain macrostructural volume, microstructural integrity, white matter hyperintensity, cerebral small vessel disease, and cognitive function among hyperuricemic populations. Tables S6. Serum uric acid variability and brain microstructural integrity in brain subregions at the voxel-wise level. Tables S7. Association between meaningful neuroimaging markers and cognitive function.

## Data Availability

Clinical data will be available for other research groups whose proposed use of the data has been approved by an independent review committee identified for this purpose. Requests for data should be directed to the principal investigator, Dr. Zhenchang Wang (cjr.wzhch@vip.163.com).

## Abbreviations

3D: Three-dimensional
AD: Axial diffusivity
BG-EPVS: Basal ganglia enlarged perivascular space
BRAVO: Brain volume
CI: Confidence interval
CMB: Cerebral microbleed
CSF: Cerebrospinal fluid
CSVD: Cerebral small vessel disease
DTI: Diffusion tensor imaging
DWMH: Deep white matter hyperintensity
FA: Fractional anisotropy
FLAIR: Fluid-attenuated inversion recovery
GM: Gray matter
KLS: Kailuan Study
MD: Mean diffusivity
META-KLS: Multi-modality MEdical imaging sTudy bAsed on KLS
MoCA: Montreal Cognitive Assessment
MRI: Magnetic resonance imaging
PWMH: Periventricular white matter hyperintensity
RD: Radial diffusivity
SUA: Serum uric acid
T1WI: T1-weighted imaging
TFCE: Threshold-free cluster enhancement
TIV: Total intracranial volume
WM: White matter
WMH: White matter hyperintensity
