# Supplementary material for: Associations of serum uric acid variability with neuroimaging metrics and cognitive decline: a population-based cohort study
Source: BMC Med. 2024 Jun 20;22:256. doi: 10.1186/s12916-024-03479-9 (PMC11188528; doi:10.1186/s12916-024-03479-9)
Supplement: Supplementary file 1 — Additional file 1. Supplementary Methods. [file 12916_2024_3479_MOESM1_ESM.docx]

**Additional File 1. Supplementary Methods**

**Brief description of the Multi-modality MEdical imaging sTudy bAsed on KaiLuan Study**

The Kailuan Study (KLS) is an ongoing prospective occupational cohort study conducted in the Kailuan community of Tangshan, Northern China, a large, modern industrial city southeast of Beijing. Adults aged 18–98 years have been enrolled since 2006. Data from demographic questionnaires and clinical and laboratory examinations were prospectively collected every 2 years from 11 local hospitals. This cohort was designed to explore the risk factors for the development or progression of common diseases, including cardiovascular or cerebrovascular disease, diabetes, metabolic syndrome, ageing, and lifestyle diseases. Based on this population-based cohort, multiple factors related to brain health in participants over a wide age range were able to be analyzed.

State-of-the-art multi-modality medical imaging in a large population sample with a wide age range is essential for clinicians and researchers. Since December 2020, we have retrospectively and prospectively recruited participants undergoing multi-modality medical imaging examination based on the KLS. Theoretically, there was no limitation on the number of participants who enrolled in the medical imaging study based on the KLS. We aimed to enrol at least 1,000 participants in this cohort.

**Measurements and covariates**

Data from demographic characteristics and clinical and laboratory examinations were collected during each visit. Clinical history was evaluated by trained doctors during follow-up hospital visits. Blood samples were collected after fasting overnight in the morning. The uricase method was used for serum uric acid measurement over the entire follow-up time. History of hypertension was defined as a self-reported history of clinical diagnosis of hypertension, use of antihypertensive medication, or systolic blood pressure ≥ 140 mmHg or diastolic blood pressure ≥ 90 mmHg during any visit. History of diabetes was defined as a self-reported history of clinical diagnosis of diabetes, use of antidiabetic medication, or fasting blood glucose level ≥ 7.0 mmol/L during any visit. Sex, smoking habits, habitual alcohol consumption, physical activity, and history of hypertension or diabetes were categorical variables, while the other covariates (age, body mass index, total cholesterol, triglyceride, high-density lipoprotein cholesterol, and low-density lipoprotein cholesterol) were continuous variables that were calculated as time-weighted average values (except for age). “Usually performing physical activity” was defined as more than three times a week for more than 30 min each time.
