# Supplementary material for: Associations of serum uric acid variability with neuroimaging metrics and cognitive decline: a population-based cohort study
Source: BMC Med. 2024 Jun 20;22:256. doi: 10.1186/s12916-024-03479-9 (PMC11188528; doi:10.1186/s12916-024-03479-9)
Supplement: Supplementary file 2 — Additional file 2: Figures S1-S2. Fig. S1. Schematic diagram of the study. Fig. S2. Flowchart of included participants. [file 12916_2024_3479_MOESM2_ESM.docx]

**Additional File 2**

**Figure S1. Schematic diagram of the study**


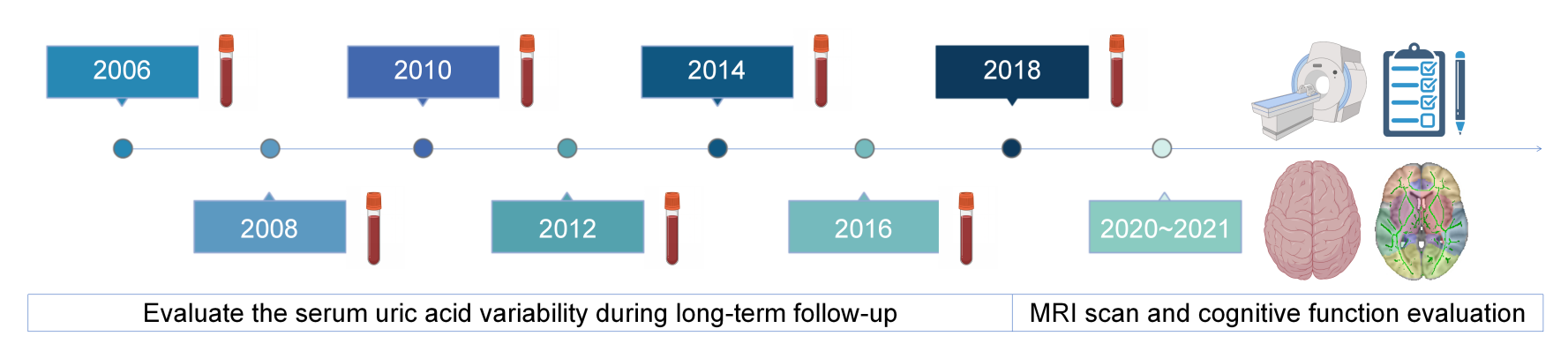


Abbreviation: MRI, magnetic resonance imaging

**Figure S2. Flowchart of included participants**


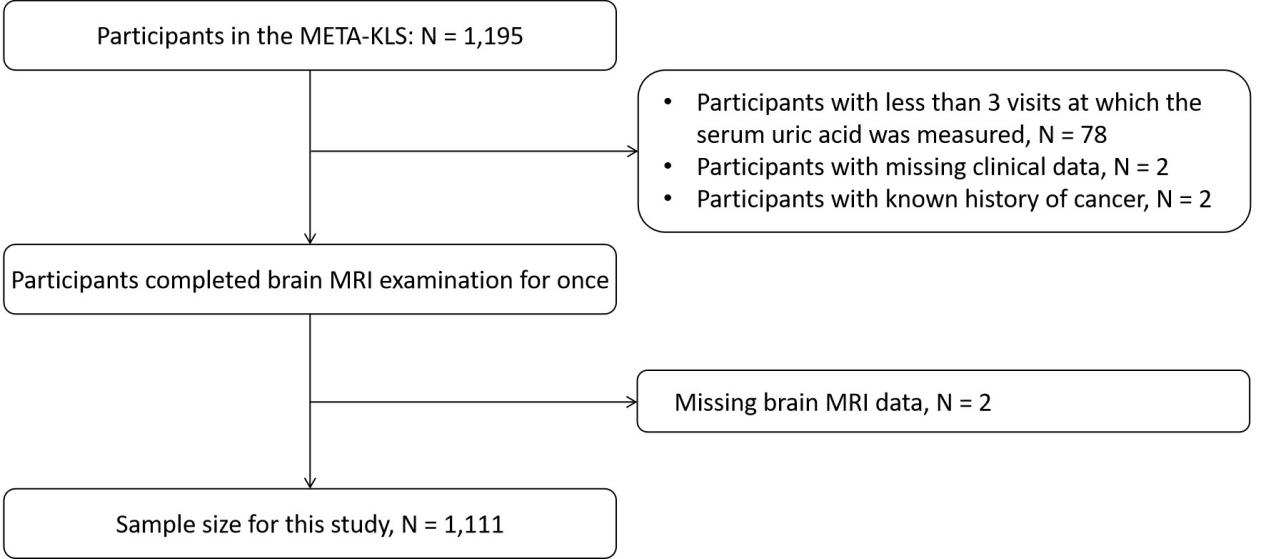


Abbreviations: META-KLS, Multi-modality MEdical imaging sTudy bAsed on KaiLuan Study; MRI, magnetic resonance imaging
