# Supplementary material for: Associations of serum uric acid variability with neuroimaging metrics and cognitive decline: a population-based cohort study
Source: BMC Med. 2024 Jun 20;22:256. doi: 10.1186/s12916-024-03479-9 (PMC11188528; doi:10.1186/s12916-024-03479-9)
Supplement: Supplementary file 3 — Additional file 3: Tables S1-S7. Tables S1. Detailed neuroimaging scanning parameters. Tables S2. Serum uric acid variability, brain macrostructural volume, microstructural integrity, white matter hyperintensity, cerebral small vessel disease, and cognitive function stratified by age. Tables S3. Serum uric acid variability, brain macrostructural volume, microstructural integrity, white matter hyperintensity, cerebral small vessel disease, and cognitive function stratified by sex. Tables S4. Serum uric acid variability, brain macrostructural volume, microstructural integrity, white matter hyperintensity, cerebral small vessel disease, and cognitive function among non-hyperuricemic populations. Tables S5. Serum uric acid variability, brain macrostructural volume, microstructural integrity, white matter hyperintensity, cerebral small vessel disease, and cognitive function among hyperuricemic populations. Tables S6. Serum uric acid variability and brain microstructural integrity in brain subregions at the voxel-wise level. Tables S7. Association between meaningful neuroimaging markers and cognitive function. [file 12916_2024_3479_MOESM3_ESM.docx]

**Additional File 3**

**Table S1. Detailed neuroimaging scanning parameters**

DTI, diffusion tensor imaging; 3D-BRAVO T1WI, three-dimensional brain volume T1-weighted imaging; FLAIR, fluid-attenuated inversion recovery; SWAN, susceptibility-weighted angiography; T2WI, T2-weighted imaging; DWI, diffusion-weighted imaging; FSE, fast spin echo; GRE, gradient-recalled echo; EPI, echo-planar imaging; TR, repetition time; TE, echo time; FOV, field of view; ETL, echo train length; NEX, number of excitations.

**Table S2. Serum uric acid variability, brain macrostructural volume, microstructural integrity, white matter hyperintensity, cerebral small vessel disease, and cognitive function stratified by age**

^a^Covariates included age, sex, smoking habits, habitual alcohol consumption, physical activity routines, body mass index, history of hypertension, history of diabetes, total cholesterol, triglyceride, high-density lipoprotein cholesterol, and low-density lipoprotein cholesterol.

^b^With further adjustment for white matter and white matter hyperintensity volume in addition to the covariates.

^c^With further adjustment for the proportion of white matter hyperintensity volume to total intracranial volume.

**Table S3. Serum uric acid variability, brain macrostructural volume, microstructural integrity, white matter hyperintensity, cerebral small vessel disease, and cognitive function stratified by sex**

^a^Covariates included age, sex, smoking habits, habitual alcohol consumption, physical activity routines, body mass index, history of hypertension, history of diabetes, total cholesterol, triglyceride, high-density lipoprotein cholesterol, and low-density lipoprotein cholesterol.

^b^With further adjustment for white matter and white matter hyperintensity volume.

^c^With further adjustment for the proportion of white matter hyperintensity volume to total intracranial volume.

**Table S4. Serum uric acid variability, brain macrostructural volume, microstructural integrity, white matter hyperintensity, cerebral small vessel disease, and cognitive function among non-hyperuricemic populations**

^a^Covariates included age, sex, smoking habits, habitual alcohol consumption, physical activity routines, body mass index, history of hypertension, history of diabetes, total cholesterol, triglyceride, high-density lipoprotein cholesterol, and low-density lipoprotein cholesterol.

^b^With further adjustment for white matter and white matter hyperintensity volume.

^c^With further adjustment for the proportion of white matter hyperintensity volume to total intracranial volume.

**Table S5. Serum uric acid variability, brain macrostructural volume, microstructural integrity, white matter hyperintensity, cerebral small vessel disease, and cognitive function among hyperuricemic populations**

^a^Covariates included age, sex, smoking habits, habitual alcohol consumption, physical activity routines, body mass index, history of hypertension, history of diabetes, total cholesterol, triglyceride, high-density lipoprotein cholesterol, and low-density lipoprotein cholesterol.

^b^With further adjustment for white matter and white matter hyperintensity volume.

^c^With further adjustment for the proportion of white matter hyperintensity volume to total intracranial volume.

**Table S6. Serum uric acid variability and brain microstructural integrity in brain subregions at the voxel-wise level**


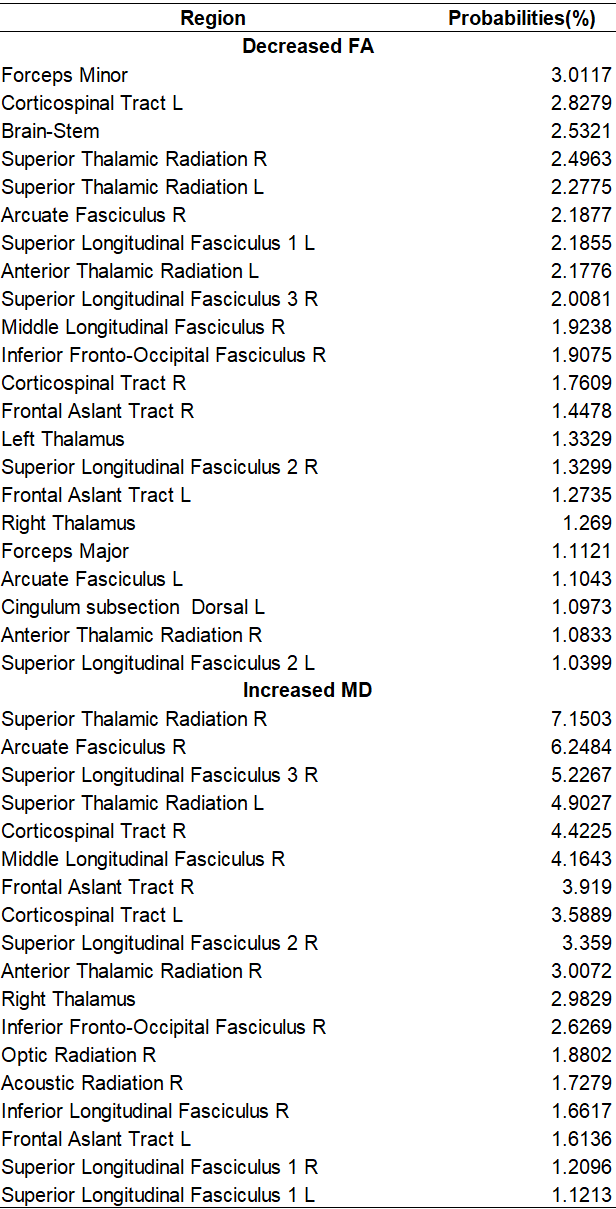


(Continued table)


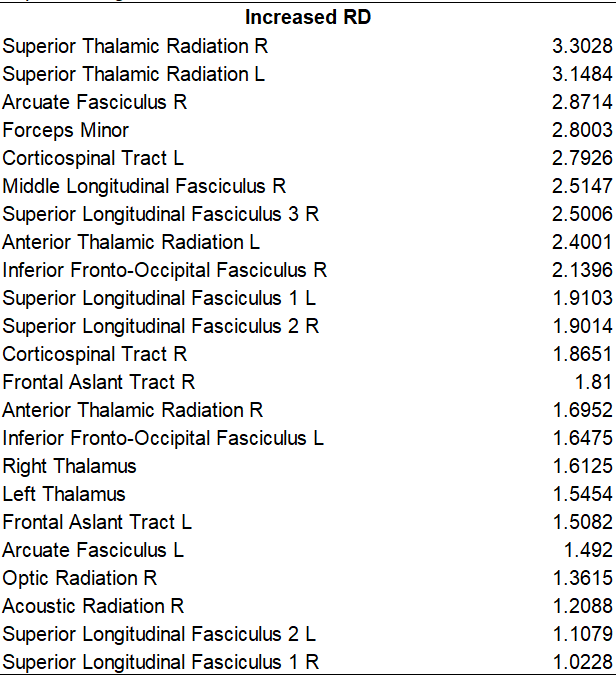


Abbreviations: FA, fractional anisotropy; MD, mean diffusivity; RD, radial diffusivity; L, left; R, right.

Clusters with *P*_threshold-free cluster enhancement_ < 0.01 with probabilities of affected tracts threshold >1% are reported. Probabilities of the affected tracts gives the (average) probability of all significant voxels that are a member of the different labelled regions within the atlas (XTRACT HCP Probabilistic Tract Atlases and Harvard-Oxford Subcortical Structural Atlas), calculated using the FSL tool ‘atlasquery’.

**Table S7. Association between meaningful neuroimaging markers and cognitive function.**

Covariates included age, sex, smoking habits, habitual alcohol consumption, physical activity routines, body mass index, history of hypertension, history of diabetes, total cholesterol, triglyceride, high-density lipoprotein cholesterol, and low-density lipoprotein cholesterol.
